# Suicidal behaviour in huntington disease

**DOI:** 10.1192/j.eurpsy.2021.1560

**Published:** 2021-08-13

**Authors:** R. Mota Freitas, M.T. Valadas

**Affiliations:** 1 Departamento De Psiquiatria E Saúde Mental, Hospital do Espírito Santo de Évora, Évora, Portugal; 2 Serviço De Psiquiatria, Unidade Local de Saúde do Baixo Alentejo, Beja, Portugal

**Keywords:** Huntington Disease, Suicide

## Abstract

**Introduction:**

Huntington Disease (HD) is a genetic, progressive neurodegenerative disorder. Its clinical features include motor dysfunction, cognitive impairments, and psychiatric symptoms. The association between HD and suicide is well documented, and the risk of suicide in HD is higher than in patients with other neurological diseases.

**Objectives:**

We aim to review the literature regarding suicidal behaviour in HD.

**Methods:**

We performed an updated review in the PubMed database using the terms “suicide”, “suicidal behaviour” and “Huntington Disease”. The included articles were selected by title and abstract.

**Results:**

The most relevant risk factors associated with suicidality in HD are depression, anxiety, and aggression, so the presence of psychiatric diagnoses should be closely monitored. No consistent results have been found regarding gender. Evidence for periods of elevated risk of suicidal behaviour in HD is mixed and the data on specific pharmacological interventions for alleviating suicidal ideation in HD is scarce.

**Conclusions:**

Patients with HD are at a high risk for suicide. This risk is further increased when a comorbid psychiatric disorder is present. It is important for the practicing psychiatrist to be aware of this association to correctly manage patients with HD, thus helping prevent suicidal behaviour.

